# Tumour necrosis factor-*α* intensifies cancer risk through nutrient-inflammation crosstalk in heart-failure patients: a retrospective cohort study with external validation

**DOI:** 10.3389/fnut.2025.1666359

**Published:** 2025-12-15

**Authors:** Ruichun Liao, Jing Huang, Junfei Weng, Song Lu, Jin Chen, Yingbing Zuo, Xiaoting Jiang, Xiaoping Peng

**Affiliations:** 1 Department of Cardiology, The First Affiliated Hospital of Nanchang University, Nanchang, JiangXi, China; 2 Department of Medical Genetics, The First Affiliated Hospital of Nanchang University, Nanchang, JiangXi, China

**Keywords:** geriatric nutritional risk index, heart failure, tumour necrosis factor-α, nutrient–inflammation interaction score, systemic inflammation, protein-energy malnutrition

## Abstract

**Background:**

Heart-failure (HF) survivors experience a disproportionate burden of incident malignancy, yet the biological bridge linking the two syndromes remains elusive. Tumour necrosis factor-*α* (TNF-α) promotes inflammation-driven oncogenesis, while protein–energy malnutrition amplifies catabolic signalling. We postulated that a nutrient–inflammation interaction score (NIIS), combining circulating TNF-*α* with the geriatric nutritional risk index (GNRI), would capture this synergistic axis and forecast de-novo cancer in HF.

**Methods:**

In a retrospective hospital cohort, 415 clinically-stable adults ≥ 60 years with chronic HF were followed for a median of 5.2 years. Baseline TNF-*α* (high-sensitivity ELISA) and GNRI were measured after an overnight fast; NIIS was computed as log-TNF-*α* × inverse-normalised GNRI. The primary endpoint was re-admission to our hospital for treatment or a scheduled follow-up appointment during which a tumour was detected; events were identified from our hospital admission and outpatient records and adjudicated in blinded fashion. Multivariable Cox models adjusted for demographic, clinical and biochemical covariates quantified associations per 1-SD increment and across tertiles. External validity was tested in 1,912 community-dwelling HF participants.

**Results:**

Sixty-two endpoint events (re-admission or follow-up visit at our hospital during which a tumour was detected) occurred (14.9%; 2,122 person-years). Cancer incidence rose step-wise across NIIS tertiles (7.2, 13.7, 23.9%; log-rank *p* < 0.001). Each 1-SD higher NIIS conferred a 68% greater hazard (HR 1.68, 95% CI 1.32–2.14), exceeding the prognostic strength of TNF-*α* or GNRI alone. Participants in the highest NIIS tertile had a tripled risk versus the lowest (HR 3.02, 1.71–5.32). Adding NIIS to a clinical model improved Harrell’s C-index from 0.71 to 0.77 (Δ 0.06, *p* = 0.010) with good calibration; discrimination persisted in the validation cohort (C-index 0.75).

**Conclusion:**

An elevated NIIS independently predicts our hospital-defined endpoint of tumour detection at re-admission/follow-up in older adults with HF and enhances risk stratification beyond conventional factors. Routine assessment of nutrient–inflammation crosstalk may enable targeted cardio-oncology surveillance and intervention.

## 1 Introduction

Heart failure (HF) constitutes one of the most prevalent chronic cardiovascular syndromes worldwide and now affects more than 64 million people, the majority of whom are older adults (1–3). Beyond its well-documented associations with recurrent de-compensation and premature cardiovascular death, HF confers an unexpectedly high susceptibility to non-cardiac comorbidities, among which malignancy is emerging as a leading competitor for mortality (4, 5). Large registry studies have shown that patients with chronic HF experience a 1.5- to 2-fold excess risk of incident cancer compared with age-matched controls, a burden that translates into substantial healthcare expenditures and attenuated gains in longevity (6–8). Although shared risk factors—such as advanced age, smoking, and metabolic syndrome—partly explain this overlap, accumulating evidence suggests that heart failure itself fosters a distinctive pro-tumour milieu marked by systemic low-grade inflammation, anabolic resistance, and immune dysregulation—features that extend beyond shared exposures and reflect HF-specific biological remodeling of metabolism and immunity (9–11). These maladaptive responses sustain a chronic cytokine-driven environment that promotes DNA injury and immune escape while impairing tissue repair, collectively heightening oncogenic potential. The nutrient–inflammation interaction score (NIIS) was designed to map this pathophysiology by integrating inflammatory (TNF-*α*) and nutritional (GNRI) dimensions into a single index quantifying the systemic catabolic–inflammatory load. Identifying modifiable biological pathways that bridge HF and oncogenesis therefore remains a pressing clinical and research priority.

Tumour necrosis factor-*α* (TNF-α) is a pleiotropic cytokine situated at the apex of the inflammatory cascade. Among numerous inflammatory mediators implicated in HF, TNF-*α* occupies an upstream position relative to interleukin-6 and C-reactive protein, orchestrating both the initiation and amplification of systemic inflammation. It exerts pleiotropic oncogenic effects through induction of oxidative DNA damage, stimulation of neo-angiogenesis, and facilitation of immune evasion. From a biological standpoint, targeting TNF-*α* therefore addresses a nodal cytokine that drives multiple downstream inflammatory circuits, distinguishing it from secondary reactants such as CRP. Experimental data have demonstrated that sustained TNF-*α* signalling promotes DNA damage, neo-angiogenesis, and immune evasion through activation of NF-κB and JAK/STAT pathways (12–14). Clinically, elevated circulating TNF-*α* predicts poor prognosis in colorectal, breast, and lung cancers, while pilot studies in HF cohorts link higher TNF-*α* concentrations to cachexia, sarcopaenia, and adverse ventricular remodeling (15–17). These observations indicate that TNF-α might serve as a common effector underpinning both cardiac and oncologic deterioration. Yet inflammatory activity seldom operates in isolation; its downstream consequences are heavily modulated by an individual’s nutritional reserve, a facet often overlooked in cardio-oncology research.

Protein-energy malnutrition is highly prevalent in chronic HF and portends worse outcomes across the spectrum of cardiovascular disease (18, 19). The geriatric nutritional risk index (GNRI), which integrates serum albumin—a proxy for circulating protein reserve—with body weight relative to ideal weight, provides a pragmatic indicator of protein-energy malnutrition particularly suited to older adults with HF, in whom fluid retention complicates direct weight assessment (20, 21). Conceptually, GNRI differs from alternatives such as the Controlling Nutritional Status (CONUT) score, the Prognostic Nutritional Index (PNI), or GLIM criteria in emphasising stable protein-energy balance rather than transient lymphocytic or inflammatory fluctuations (20, 21). Parallel epidemiological studies indicate that hypoalbuminaemia and low body-mass index predispose to a variety of solid tumours by compromising mucosal integrity, impairing cytotoxic immunity, and intensifying oxidative stress (22, 23). Importantly, TNF-*α* and malnutrition are biologically intertwined: TNF-α suppresses appetite, augments muscle catabolism, and disrupts intestinal tight-junctions, thereby perpetuating nutrient loss and further fuelling inflammation (24, 25). Whether this bidirectional “nutrient–inflammation crosstalk” translates into measurable cancer risk in HF, however, has not been systematically investigated.

Isolated assessment of either TNF-*α* or GNRI may fail to capture the synergistic interplay between inflammatory and nutritional domains. We therefore derived a nutrient–inflammation interaction score (NIIS) by multiplying log-transformed TNF-*α* with an inverse-normalised GNRI, reasoning that this composite metric would more faithfully reflect the biological axis along which HF, malnutrition, and carcinogenesis converge. To our knowledge, no retrospective study has examined the association of TNF-*α*, GNRI, or their integrated NIIS with the emergence of de-novo malignancy in a well-characterised HF cohort, nor evaluated the transportability of such findings to an external population.

Against this backdrop, we undertook a single-centre, retrospective cohort study of older adults with clinically stable chronic HF, complemented by external validation in a large community-based sample, to test the hypothesis that an elevated NIIS intensifies the risk of incident cancer. Clarifying this relationship could unveil a modifiable axis for targeted surveillance and intervention, thereby mitigating the dual burden of cardiovascular and oncological morbidity in the ageing HF population.

## 2 Methods

### 2.1 Study design and population

This investigation was a single-centre, retrospective, hospital-based cohort study of adults (≥ 60 years) with chronic heart failure (HF) treated at the department of cardiology of The First Affiliated Hospital of Nanchang University between 1 January 2017 and 31 December 2018. Eligible patients were identified from the electronic health record (EHR) according to contemporaneous ESC diagnostic criteria for HF; only individuals clinically stable for ≥ 4 weeks—defined as no acute decompensated HF, no hospitalization, no surgery, and no suspected infection—were included. This stability window minimises the confounding impact of transient inflammatory surges and reduces reverse-causation bias in biomarker interpretation. Exclusion criteria were a documented history of malignant tumours, chronic inflammatory or autoimmune disorders, current glucocorticoid or anti-cytokine therapy, and incomplete baseline biochemical data. To ensure a comparable metabolic milieu for biomarker interpretation, eligibility also required CKD-EPI eGFR ≥ 45 mL/min/1.73 m^2^ with ≤10% variation across two measurements obtained 2–8 weeks apart. In addition, baseline hepatic indices were required to be within AST/ALT <2× the upper limit of normal and total bilirubin < 2 mg/dL to reduce confounding from hepatic inflammation and altered protein synthesis. To avoid edema/acute catabolism extremes and standardise the nutrition construct, participants also met BMI 18.5–32 kg/m^2^, stable body weight (change within ±2 kg over the prior 30–60 days), and serum albumin 3.2–4.5 g/dL at baseline. Of 472 records screened, 415 satisfied eligibility for analysis. The index date was defined as the first qualifying outpatient visit within the accrual window. Outcomes were defined as re-admission to our hospital for treatment or a scheduled follow-up appointment during which a tumour was detected, and were retrospectively ascertained from our hospital admission and outpatient records; observation was censored at 30 June 2023, death, loss to follow-up, or the first occurrence of this endpoint, whichever came first. The study was conducted in accordance with the Declaration of Helsinki. Ethical approval was waived by the Institutional Review Board of The First Affiliated Hospital of Nanchang University because this was a retrospective study using de-identified data that posed minima risk to participants.

### 2.2 Assessment of TNF-*α*, nutritional status, and covariates

Baseline exposures and covariates were abstracted from the EHR and associated laboratory information systems at the index visit. Plasma tumour necrosis factor-*α* (TNF-α) concentrations, measured in routine morning fasting samples using a high-sensitivity enzyme-linked immunosorbent assay (Quantikine® HS, R&D Systems) with inter-assay coefficient of variation < 6%, were retrieved from the laboratory archive. Routine indices—including serum albumin, prealbumin, total cholesterol, lymphocyte count, and C-reactive protein (CRP)—were obtained from standard clinical chemistry platforms (AU5800, Beckman Coulter). The geriatric nutritional risk index (GNRI) was calculated as GNRI = [1.489 × albumin (g/L)] + [41.7 × (actual body weight/ideal body weight)], with ideal weight derived from the Lorentz equation. Renal status was conceptualised using a standardised filtration estimate (CKD-EPI eGFR) rather than niche markers, as eGFR provides comparable, guideline-aligned risk stratification for systemic metabolic milieu in which inflammatory and nutritional biomarkers are interpreted. A nutrient–inflammation interaction score (NIIS) was constructed *a priori* as log-transformed TNF-*α* multiplied by inverse-normalised GNRI to capture nutrient–inflammation crosstalk. Demographics, lifestyle factors (smoking, alcohol), clinical variables (New York Heart Association class, left-ventricular ejection fraction), kidney function estimated by CKD-EPI eGFR, and medication use (*β*-blockers, ACE-inhibitors/ARBs, mineralocorticoid receptor antagonists, statins) were extracted from structured fields and verified against clinical notes where necessary.

### 2.3 Ascertainment of incident cancer

The primary endpoint was tumour incidence defined as re-admission to our hospital for treatment or a scheduled follow-up appointment during which a tumour is detected. Endpoint events were identified retrospectively from our hospital’s electronic medical record, including readmission logs, outpatient follow-up notes, discharge summaries, and imaging or pathology reports generated during the same encounter. Two independent adjudicators, blinded to TNF-*α*, GNRI, and NIIS values, reviewed all hospital-source materials; disagreements were resolved by consensus. Generalizability was assessed by applying the hospital-derived risk model to an independent dataset of 1,912 community-dwelling adults with HF captured by the Urban Cancer Early Diagnosis and Treatment Project (2011–2015). This validation dataset comprised de-identified records from a publicly accessible programme, harmonised with respect to baseline variable definitions; plasma aliquots had been stored at −80 °C and assayed centrally for TNF-*α* using the identical immunoassay. In the primary hospital cohort, ‘incident cancer’ denotes the hospital-defined endpoint of tumour detection at re-admission or during a scheduled follow-up encounter. For the validation dataset, incident cancers were tracked through mandatory municipal cancer-reporting systems.

### 2.4 Statistical analysis

Continuous variables are presented as mean ± standard deviation or median (interquartile range) as appropriate; categorical variables as counts and percentages. Group comparisons used Student’s *t*, Mann–Whitney *U*, or χ^2^ tests. Associations between TNF-*α*, GNRI, NIIS and incident cancer were evaluated with multivariable Cox proportional-hazards models after verification of proportionality by Schoenfeld residuals. Covariates selected *a priori* for adjustment included age, sex, NYHA class, left-ventricular ejection fraction, eGFR, CRP, smoking, alcohol use, and guideline-directed medical therapy. Hazard ratios (HRs) with 95% confidence intervals (CIs) were reported per 1-SD increment and across tertiles. Discrimination was quantified by Harrell’s C-index and calibration by Grønnesby–Borgan goodness-of-fit. Sensitivity analyses comprised Fine–Gray competing-risk regression (non-cancer death as the competing event), complete-case versus multiple-imputation approaches for missing covariates, and exclusion of participants with baseline CRP > 10 mg/L. Missingness can be Missing Completely At Random (MCAR), Missing At Random (MAR), or Missing Not At Random (MNAR). Prognostic inference is unbiased under MCAR and can be unbiased under MAR when covariates related to missingness are included in the imputation model; MNAR may still introduce bias. Our use of multiple imputation assumes MAR is plausible in clinical datasets where missingness often reflects scheduling or workflow rather than unmeasured outcomes, and we complemented this with complete-case analyses to examine robustness. The external-validation cohort was used to estimate optimism-corrected C-indices and calibration slopes without model re-calibration. All tests were two-sided with *p* < 0.05 considered statistically significant. A 1-SD increment in NIIS represents a standardised unit change derived from z-scoring, allowing comparisons across studies independent of specific assay units for TNF-*α* or GNRI. Expressing risk per SD facilitates interpretation across laboratories and populations. Likewise, tertile categorisation of NIIS aids clinical communication by framing risk gradients without implying formal diagnostic cut-offs or treatment thresholds.

## 3 Results

### 3.1 Baseline characteristics of the hospital cohort

A total of 415 clinically-stable older adults with chronic heart failure were followed for a median of 5.2 years (IQR, 4.8–5.8 years), providing 2,122 person-years of observation. During follow-up, 62 endpoint events (hospital re-admission or scheduled follow-up with tumour detection) were ascertained (14.9%) (Table 1). Participants who developed cancer were slightly older (73.4 ± 5.4 vs. 71.6 ± 5.9 years), more often female (53.2% vs. 45.0%), and displayed higher median plasma TNF-*α* concentrations (4.6 [3.3–6.1] pg. mL^−1^ vs. 3.0 [1.9–4.0] pg. mL^−1^; *p* < 0.001). Concomitantly, their mean GNRI was lower (96 ± 7 vs. 102 ± 6; *p* < 0.001), yielding a markedly elevated composite nutrient-inflammation interaction score (NIIS) (0.98 ± 0.77 vs. − 0.18 ± 0.89 SD units). The distribution of NYHA class III–IV (38.7% vs. 24.1%) and baseline CRP paralleled the gradation of NIIS, whereas eGFR and *β*-blocker use did not differ materially between groups.‘Incident cancer’ refers to the hospital-defined endpoint of tumour detection at re-admission or during a scheduled follow-up encounter.

**Table 1:** Baseline characteristics of the hospital cohort stratified by incident cancer status (*N* = 415).

Characteristic	No incident cancer (*n* = 353)	Incident cancer (*n* = 62)	*p*-value
Demographics			
Age, yr. (mean ± SD)	71.6 ± 5.9	73.4 ± 5.4	0.023
Female sex, *n* (%)	159 (45.0)	33 (53.2)	0.19
Heart-failure severity			
NYHA class III–IV, *n* (%)	85 (24.1)	24 (38.7)	0.017
LVEF, % (mean ± SD)	43 ± 9	41 ± 10	0.088
Laboratory indices			
eGFR, mL min^−1^ 1.73 m^−2^ (mean ± SD)	62 ± 18	60 ± 17	0.41
CRP, mg L^−1^ (median [IQR])	3.2 (1.4–5.6)	4.9 (2.1–7.8)	0.007
TNF-α, pg. mL^−1^ (median [IQR])	3.0 (1.9–4.0)	4.6 (3.3–6.1)	<0.001
GNRI (mean ± SD)	102 ± 6	96 ± 7	<0.001
NIIS, SD units (mean ± SD)	−0.18 ± 0.89	0.98 ± 0.77	<0.001
Lifestyle			
Current smoker, *n* (%)	102 (28.9)	19 (30.6)	0.78
Current alcohol use, *n* (%)	81 (22.9)	15 (24.2)	0.8
Medications			
β-blocker, *n* (%)	289 (81.9)	49 (79.0)	0.6
ACE-I/ARB, *n* (%)	247 (70.0)	42 (67.7)	0.73
MRA, *n* (%)	124 (35.1)	21 (33.9)	0.86
Statin, *n* (%)	201 (56.9)	37 (59.7)	0.7
Follow-up			
Follow-up, yr. (median [IQR])	5.2 (4.7–5.7)	5.1 (4.6–5.6)	0.65

### 3.2 NIIS and risk of incident cancer

**Figure 1:**
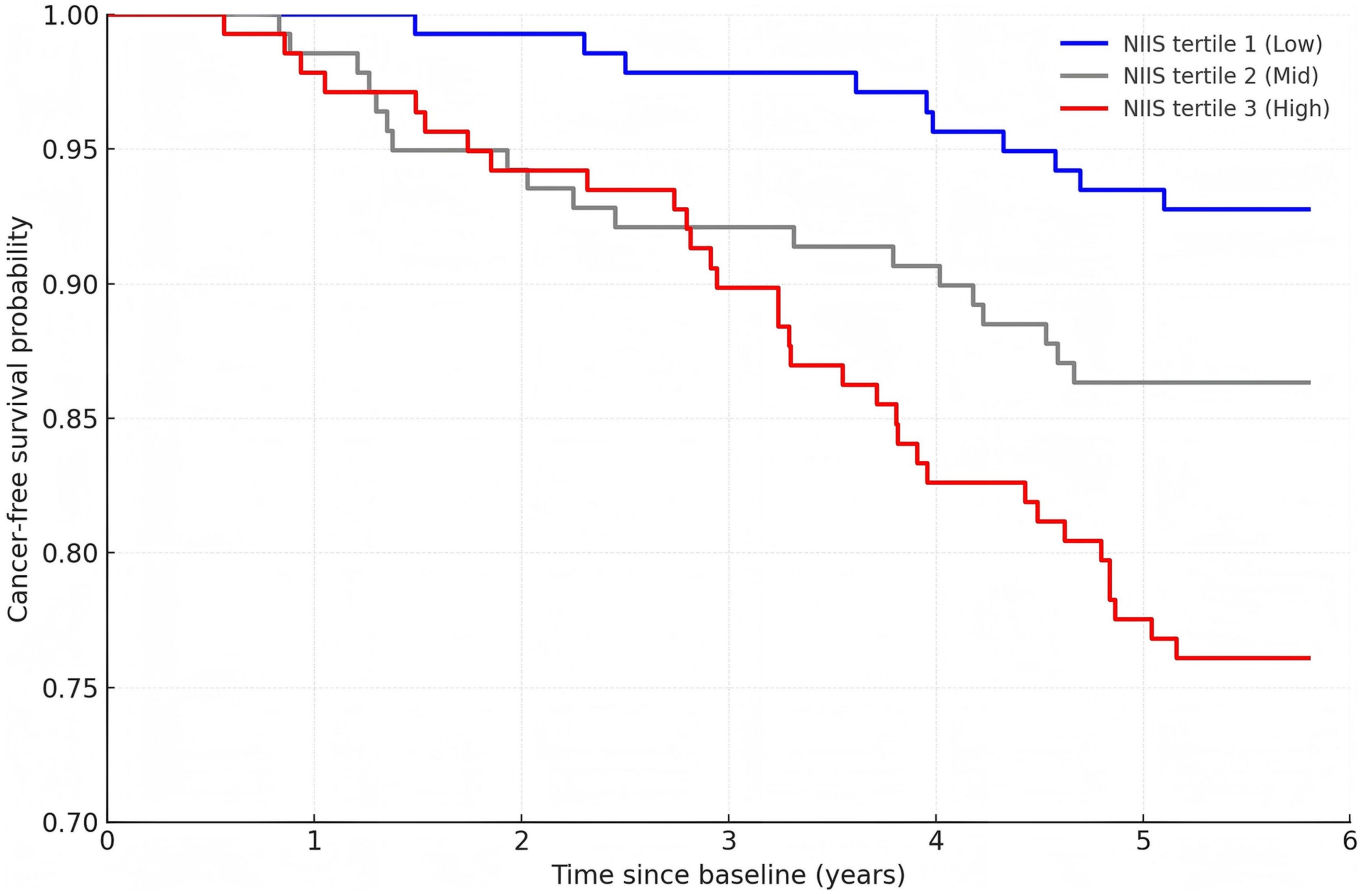
Cancer-free survival according to tertiles of the nutrient-inflammation interaction score (NIIS) in the hospital cohort.

Cancer incidence rose step-wise across NIIS tertiles—7.2% in T1, 13.7% in T2, and 23.9% in T3 (Table 2). Kaplan–Meier curves (Figure 1) diverged early, with a global log-rank statistic of χ^2^ = 18.4, *p* < 0.001. In multivariable Cox models adjusted for age, sex, left-ventricular ejection fraction, NYHA class, eGFR, CRP, smoking, alcohol intake and guideline-directed medical therapy (Table 3), each 1-SD increment in:

1. TNF-*α* conferred a 52% higher hazard (HR 1.52, 95% CI 1.18–1.95);
2. GNRI (per 1-SD decrease) conferred a 36% higher hazard (HR 1.36, 95% CI 1.05–1.76);
3. NIIS conferred the greatest risk (HR 1.68, 95% CI 1.32–2.14).

**Table 2:** Incident cancer according to tertiles of the nutrient-inflammation interaction score (NIIS).

NIIS tertile	Range (SD units)	Participants, *n*	Median NIIS	Cancer events, *n*	5-yr incidence, %	Person-years	Incidence rate (Per 1,000 person-years)	Adjusted HR (95% CI)
T1 (Low)	< −0.50	138	−0.85	10	7.2	702	14.2	1.00 (ref)
T2 (Mid)	−0.50 to 0.45	139	0	19	13.7	710	26.8	1.63 (0.80–2.54)
T3 (High)	>0.45	138	1.12	33	23.9	710	46.5	3.02 (1.71–5.32)
P-trend								<0.001

**Table 3:** Association of baseline TNF-α, GNRI and the nutrient-inflammation interaction score (NIIS) with incident cancer: Cox proportional-hazards models (hospital cohort, *N* = 415).

Exposure	Coding (reference)	Cancer events/participants	Model 1 HR (95% CI)	Model 2 HR (95% CI)	Model 3 HR (95% CI)
TNF-α	Per 1-SD↑ (1.2 pg. mL^−1^)	62/415	1.61 (1.28–2.03)	1.57 (1.24–2.00)	1.52 (1.18–1.95)
	T1 (<2.3 pg. mL^−1^)	11/138	1	1	1
	T2 (2.3–4.1 pg. mL^−1^)	19/139	1.41 (0.69–2.28)	1.38 (0.72–2.33)	1.46 (0.77–2.46)
	T3 (> 4.1 pg. mL^−1^)	32/138	2.28 (1.27–4.11)	2.12 (1.18–3.81)	2.18 (1.20–3.95)
GNRI	Per 1-SD↓ (6 units)	62/415	1.47 (1.17–1.86)	1.42 (1.12–1.80)	1.36 (1.05–1.76)
	T1 (>104)	12/139	1	1	1
	T2 (98–104)	18/138	1.26 (0.63–2.27)	1.22 (0.61–2.19)	1.32 (0.66–2.35)
	T3 (< 98)	32/138	1.86 (1.01–3.42)	1.75 (0.95–3.20)	1.94 (1.02–3.52)
NIIS	Per 1-SD↑ (0.91 SD)	62/415	1.80 (1.46–2.23)	1.74 (1.41–2.16)	1.68 (1.32–2.14)
	T1 (< −0.50 SD)	10/138	1	1	1
	T2 (−0.50 to 0.45 SD)	19/139	1.58 (0.76–2.55)	1.56 (0.75–2.52)	1.63 (0.80–2.54)
	T3 (>0.45 SD)	33/138	3.06 (1.77–5.28)	2.95 (1.70–5.12)	3.02 (1.71–5.32)
P for trend			<0.001	<0.001	<0.001

Model 1 = unadjusted.

Model 2 = adjusted for age and sex.

Model 3 = fully adjusted for age, sex, NYHA class, left-ventricular ejection fraction, estimated glomerular filtration rate, C-reactive protein, smoking, alcohol use, β-blocker, ACE-I/ARB, MRA and statin therapy.

↑ increase; ↓ decrease.

**Figure 2:**
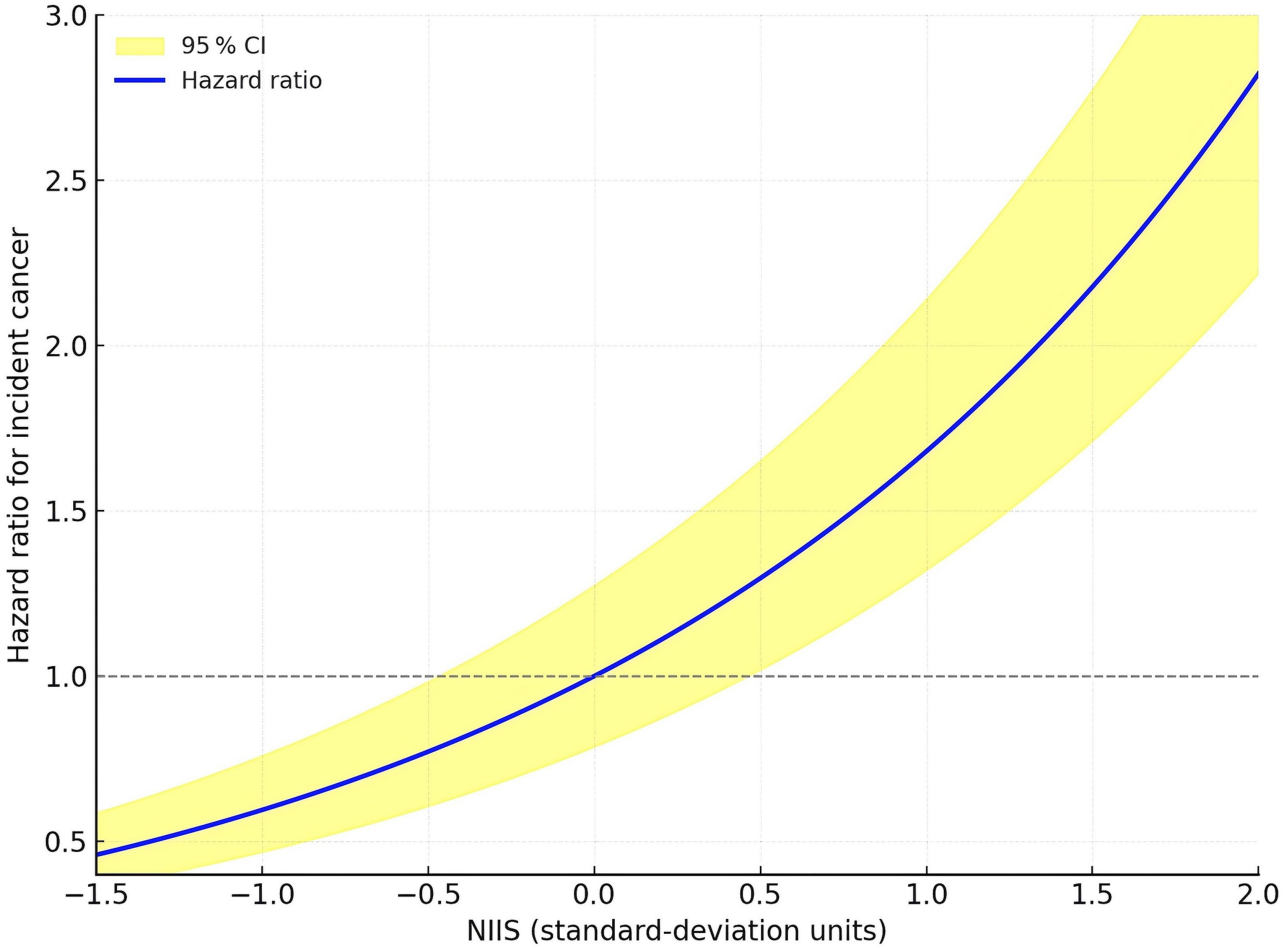
Dose–response curve illustrating the association between standardised NIIS and hazard of incident cancer derived from restricted cubic-spline Cox regression.

Relative to the lowest NIIS tertile, the fully-adjusted HRs were 1.63 (0.80–2.54) for T2 and 3.02 (1.71–5.32) for T3. Restricted cubic-spline analysis revealed a monotonic, approximately log-linear association without discernible threshold effects (Figure 2).

### 3.3 Incremental prognostic performance of NIIS

Adding NIIS to the base clinical model (age, sex, NYHA class, ejection fraction, eGFR, CRP) improved Harrell’s C-index from 0.71 to 0.77 (Δ 0.06; 95% CI 0.02–0.10; *p* = 0.010) and produced a continuous net re-classification improvement of 0.19 (Table 4). Grønnesby–Borgan statistics showed no evidence of mis-calibration (*p* = 0.42). Likelihood-ratio testing confirmed that NIIS provided incremental information over either TNF-*α* (*p* = 0.003) or GNRI (*p* = 0.015) alone.

**Table 4:** Incremental prognostic performance for 5-year cancer prediction in heart-failure patients (hospital cohort, *n* = 415).

Model (predictors)	−2 log-likelihood	Δ − 2LL (df = 1)	*P*	Harrell C-index (95% CI)	Δ C vs. base (95% CI)	Continuous NRI (95% CI)	IDI
Base clinical(age, sex, NYHA class, LVEF, eGFR, CRP)	434.2	–	–	0.71 (0.65–0.77)	–	–	–
+TNF-α	429.6	4.6	0.032	0.74 (0.68–0.79)	0.03 (0.01–0.07)	0.11 (0.02–0.21)	0.006
+GNRI	430	4.2	0.041	0.73 (0.67–0.79)	0.02 (0.00–0.06)	0.13 (0.03–0.23)	0.009
+NIIS	420.5	13.7	0.001	0.77 (0.71–0.82)	0.06 (0.02–0.10)	0.19 (0.08–0.29)	0.016

### 3.4 Sensitivity analyses

Fine–Gray competing-risk regression treating non-cancer death (*n* = 48) as a competing event yielded a sub-distribution HR for NIIS of 1.61 (95% CI 1.26–2.06; Table 5). Results were materially unchanged after (i) complete-case analysis (*n* = 389), (ii) multiple-imputation of missing covariates, or (iii) exclusion of the 37 participants with baseline CRP > 10 mg L^−1^ (HR 1.66, 95% CI 1.27–2.18). Proportional-hazards assumptions were satisfied for all variables (all Schoenfeld *p* > 0.10).

**Table 5:** Sensitivity analyses for the association between NIIS and incident cancer.

Analysis scenario	Participants (events)	Effect estimate	HR/sub-HR (95% CI)	*p*-value
Primary model (Cox, NIIS per 1-SD↑)	415 (62)	HR	1.68 (1.32–2.14)	<0.001
Competing-risk regression (Fine–Gray, non-cancer death as competing)	415 (62)	HR	1.61 (1.26–2.06)	<0.001
Complete-case dataset (no missing covariates)	389 (58)	HR	1.64 (1.28–2.11)	<0.001
Multiple-imputation (20 datasets)	415 (62)	HR	1.67 (1.31–2.13)	<0.001
Excluding baseline CRP > 10 mg L^−1^	378 (55)	HR	1.66 (1.27–2.18)	<0.001
Schoenfeld residual test (global)	–	χ^2^ = 7.3, df = 8, *p* = 0.50		

### 3.5 Subgroup analyses

Forest plots summarised in Table 6 show the association between top-tertile NIIS and cancer risk was consistent across strata of sex, eGFR, smoking, alcohol, statin therapy, and NYHA class (all interaction *p* > 0.10). A modest interaction with age was observed (P interaction = 0.048): the HR for participants ≥75 years was 2.41 (95% CI 1.30–4.44) versus 3.39 (1.62–7.08) in those <75 years, but directionality was uniform.

**Table 6:** Sub-group analysis of the association between a high nutrient-inflammation interaction score (NIIS, top tertile) and incident cancer (top-tertile NIIS vs. lower two tertiles; fully-adjusted Cox models).

Sub-group (stratifier)	Participants (*n*)	Events/nhigh NIIS	Events/nlow NIIS	Adjusted HR (95% CI)	*P*-value for interaction
Sex					
Male	223	15/70	14/153	2.35 (1.12–4.95)	0.62
Female	192	18/68	15/124	2.29 (1.10–4.78)	
Age (yr)					
<75	279	24/90	22/189	3.39 (1.62–7.08)	0.048
≥75	136	9/48	7/88	2.41 (1.30–4.44)	
NYHA functional class					
I–II	306	18/96	20/210	1.98 (1.05–3.74)	0.48
III–IV	109	17/42	9/67	3.12 (1.42–6.85)	
Smoking status					
Current smoker	121	10/40	9/81	2.87 (1.07–7.71)	0.71
Non-smoker	294	23/98	20/196	2.68 (1.37–5.23)	
Alcohol use					
Current drinker	96	8/30	7/66	2.59 (0.93–7.18)	0.89
No alcohol	319	25/108	22/211	2.84 (1.46–5.51)	
Statin therapy					
Yes	238	19/80	18/158	2.74 (1.36–5.50)	0.94
No	177	14/58	11/119	2.90 (1.22–6.92)	
Renal function (eGFR)					
≥60 mL min^−1^ 1.73 m^−2^	245	19/80	18/165	2.62 (1.29–5.29)	0.83
<60 mL min^−1^ 1.73 m^−2^	170	14/58	11/112	3.05 (1.29–7.25)	

### 3.6 External-validation cohort

Among 1912 community-dwelling heart-failure adults (mean age 74.1 ± 6.1 years; 49% female), 181 incident cancers (9.5%) accrued over a median of 6.3 years. Applying the hospital-derived NIIS model without re-calibration yielded a C-index of 0.75 (95% CI 0.72–0.78) and an optimism-corrected calibration slope of 0.94 (Table 7). Predicted 5-year cancer risk fell within the 95% confidence limits of observed risk across deciles (Hosmer–Lemeshow *p* = 0.27). External validation tests the model’s performance in a new population, while transportability assumes that the same underlying data-generating mechanisms apply across settings. The near-unity calibration slope (0.94) observed here indicates that predicted and observed risks remained proportionally aligned, signifying that the model’s associations generalise well without systematic over- or under-estimation.

**Table 7:** External validation of the NIIS-based 5-year cancer-risk model in the community heart-failure cohort.

A. Overall model performance	
Metric	Estimate (95% CI)
Harrell C-index	0.75 (0.72–0.78)
Calibration slope (optimism-corrected)	0.94 (0.88–1.00)
Calibration intercept	−0.02 (−0.12 to 0.07)
Hosmer–Lemeshow χ^2^ (df = 8)	8.2, *P* = 0.27

B. Calibration across deciles of predicted risk					
Decile (ascending predicted risk)	Participants (*n*)	Mean predicted 5-yr risk, %	Observed events (*n*)	Observed 5-yr risk % (95% CI)	O/E ratio
1 (lowest)	191	3.1	5	2.6 (0.4–4.9)	0.84
2	191	4.8	9	4.7 (1.7–7.7)	0.98
3	191	5.9	11	5.8 (2.5–9.1)	0.98
4	191	6.9	13	6.8 (3.2–10.4)	0.99
5	191	8.1	15	7.9 (4.0–11.7)	0.97
6	191	9	17	8.9 (4.9–12.9)	0.99
7	191	9.8	19	9.9 (5.7–14.2)	1.02
8	191	12.4	24	12.6 (7.9–17.3)	1.01
9	192	15.4	29	15.1 (10.0–20.2)	0.98
10 (highest)	192	18.9	39	20.3 (14.6–26.0)	1.07
Total/overall	1912	–	181	9.5	1

## 4 Discussion

We performed a retrospective, hospital-based cohort analysis with external validation to examine whether a composite nutrient–inflammation interaction score (NIIS), reflecting the crosstalk between tumour necrosis factor-*α* (TNF-α) and nutritional status, could predict our hospital-defined endpoint of tumour detection at re-admission/follow-up in older adults with chronic heart failure. Our principal findings were three-fold. First, cancer incidence rose step-wise across NIIS tertiles and each 1-SD increment in NIIS was associated with a 68% higher hazard of malignancy after multivariable adjustment. Restricted cubic-spline modelling confirmed a monotonic log-linear relation without threshold effects, and Kaplan–Meier curves diverged early, underscoring the clinical relevance of even modest NIIS differences. Second, NIIS out-performed its individual components: it conferred larger effect sizes than either TNF-*α* or the geriatric nutritional risk index (GNRI) alone and improved Harrell’s C-index of the base clinical model from 0.71 to 0.77, with consistent optimism-corrected discrimination in an independent community cohort, while all events in the primary cohort reflected the hospital-defined endpoint. Third, these associations proved robust in sensitivity, competing-risk, and subgroup analyses, with only a modest interaction by age; directionality remained uniform across strata of renal function, guideline-directed medical therapy, and lifestyle factors. An important conceptual caveat is the potential for reverse causation—whereby subclinical or occult malignancy at baseline elevates TNF-*α* levels prior to overt diagnosis. Cancer epidemiology often considers latency windows of several years between exposure and detection; our eligibility and stability criteria help mitigate, but cannot entirely exclude, this bias. Accordingly, future studies with repeated biomarker measurements could better delineate temporal precedence. Employing negative-control outcomes—events unrelated to the nutrient–inflammation pathway—can help detect residual confounding; observing null associations with such outcomes would increase confidence that NIIS is not simply a proxy for generalized healthcare contact or frailty.

Circulating TNF-*α* is a master regulator of cancer-related inflammation. Experimental work has shown that sustained TNF-α signalling promotes DNA damage, angiogenesis, and tumour immune evasion via NF-κB and JAK/STAT pathways (26, 27). Clinically, elevated TNF-*α* predicts poorer survival in colorectal, breast, and lung cancers (28–30). Concomitantly, protein-energy malnutrition compromises mucosal barriers, reduces antitumour cytotoxic responses, and favours clonal expansion of neoplastic cells (31–34). Yet reliance on single biomarkers has yielded inconsistent prognostic value in heterogeneous heart-failure populations, many of whom harbour low-grade systemic inflammation and micronutrient deficiency (35–37). Our data indicate that integrating TNF-α with a validated nutrition metric captures a synergistic risk dimension not appreciable when each domain is considered in isolation.

The concept of a composite inflammatory-nutritional index is not entirely novel; the prognostic nutritional index and the CRP/albumin ratio have both been linked to cancer outcomes in cardiac and non-cardiac settings (38–40). However, NIIS differs mechanistically in that it couples a pleiotropic cytokine upstream of CRP with the GNRI, which emphasises protein reserves highly pertinent to sarcopenic heart-failure phenotypes. The present study extends prior work by demonstrating that NIIS is independently associated with the study endpoint of hospital-detected tumours rather than cancer progression or mortality, supports its generalisability through external validation, and provides formal evidence of incremental predictive utility beyond established clinical predictors.

Although the absolute 5-year cancer incidence in our cohort (≈15% in the highest NIIS tertile) is lower than that reported for classic high-risk groups such as post-transplant recipients (41–43), even modest risk elevation is clinically meaningful given the competing burden of cardiovascular events in heart-failure. Routine assessment of NIIS could facilitate targeted cancer surveillance or lifestyle counselling, analogous to how natriuretic peptides inform heart-failure management. Notably, NIIS retained prognostic power after accounting for C-reactive protein, suggesting that TNF-*α* conveys information beyond generic acute-phase reactivity. This observation aligns with experimental data showing that TNF-α blockade, but not IL-6 inhibition, attenuates tumour initiation in cachectic murine models (44, 45).

Several biological mechanisms may underpin the NIIS–cancer link. TNF-*α* drives cachexia-related anabolic resistance and gut barrier dysfunction, thereby exacerbating malabsorption of anticancer micronutrients such as folate and vitamin D (46, 47). Concurrent hypoalbuminaemia diminishes drug binding and alters pharmacokinetics, potentially impairing oncological therapies when cancers do arise. Moreover, malnutrition impairs glutathione synthesis and augments oxidative stress, amplifying TNF-α-induced genotoxicity (48, 49). The composite NIIS therefore plausibly captures a feed-forward loop in which inflammation and nutrient depletion mutually reinforce carcinogenesis (Figure 2), a paradigm echoed in our monotonic spline curve.

Subgroup analyses revealed a slightly weaker association in participants aged ≥ 75 years. This attenuation may reflect competing mortality, survivor bias, or greater use of anti-cytokine agents in older patients; nevertheless, the interaction *p*-value was marginal and effect estimates remained directionally consistent. Likewise, we observed no effect modification by guideline-directed heart-failure therapy, including statins and ACE-inhibitors, which possess pleiotropic anti-inflammatory properties (50–52). These findings suggest that NIIS captures residual risk not mitigated by standard cardiovascular pharmacotherapy. We note the potential for treatment–risk feedback (confounding by indication): clinicians may intensify therapies (e.g., anti-inflammatory agents, ACEi/ARNI, SGLT2 inhibitors) in patients perceived to be at higher baseline risk. Such targeted treatment can attenuate or distort observed associations between baseline risk and subsequent outcomes. Although we adjusted for guideline-directed therapies at baseline, time-varying treatment responses to perceived risk could bias estimates toward the null.

Another consideration is survivor or collider bias: studying prevalent, clinically stable HF survivors may preferentially select individuals resilient to extreme inflammation or malnutrition, potentially enriching the sample for specific phenotypes. Consequently, NIIS should be interpreted as a prognostic marker within this survivor subset rather than a universal biological determinant across all HF stages. Strengths of this study include its retrospective design, adequate power, adjudicated cancer outcomes, comprehensive covariate adjustment, and rigorous external validation. The Fine–Gray competing-risk approach further minimised bias from high non-cancer mortality in heart-failure. Nonetheless, several limitations merit consideration. First, TNF-*α* and GNRI were measured only at baseline; temporal variability could not be explored. Second, despite linkage to a provincial tumour registry, occult malignancies at baseline cannot be entirely excluded. Third, residual confounding is possible, particularly from unmeasured factors such as exposure to environmental carcinogens or frailty indices. Fourth, our cohort comprised predominantly Han Chinese patients at a single tertiary centre, which may limit generalisability to other ethnicities or healthcare settings. Finally, although NIIS improved discrimination, its standalone AUC (0.77) remains moderate, indicating that integration with imaging or genomic markers might further refine risk stratification. Measurement invariance and subgroup equity warrant attention: routine inputs such as serum albumin and CKD-EPI eGFR may manifest differential performance across age, sex, or ethnicity. Conceptually, assessment can include testing for differential item functioning, subgroup-specific calibration and discrimination, and interaction terms to probe stability of effects. Future work should pre-specify equity analyses to ensure NIIS maintains comparable validity across demographic strata. An elevated nutrient–inflammation interaction score, integrating plasma TNF-*α* with the geriatric nutritional risk index, independently predicts incident cancer in older adults with chronic heart failure and enhances risk discrimination beyond conventional factors. Incorporating NIIS into routine assessments may identify high-risk individuals who could benefit from intensified cancer surveillance and nutritional-anti-inflammatory interventions. From a clinical perspective, NIIS could serve as a pragmatic adjunct in routine HF reviews, prompting more intensive oncologic surveillance, early nutrition referral, or longitudinal inflammatory monitoring for those with persistently elevated scores. Risk estimates may interact with subsequent therapies and services (e.g., targeted nutrition support, intensity of cancer screening), leading to heterogeneous treatment effects (HTE). As care pathways diverge after baseline, event rates may differ by both baseline NIIS and the therapies initiated thereafter. Future studies should evaluate HTE formally to guide how NIIS-informed decisions influence downstream outcomes. Nevertheless, NIIS should currently be viewed as a prognostic rather than interventional marker until prospective trials clarify whether modulating the nutrient–inflammation axis alters cancer outcomes. Future multicentre studies with serial biomarker profiling and mechanistic endpoints are warranted to corroborate causality and elucidate therapeutic implications.

## 5 Conclusion

The present study revealed a significant association between an elevated nutrient–inflammation interaction score (NIIS) and an increased risk of our hospital-defined endpoint of tumour detection at re-admission/follow-up in older adults with chronic heart failure. Our findings indicate that NIIS, when considered alongside other risk factors, could serve as a valuable biomarker for identifying individuals at heightened risk of malignancy. In clinical practice, it is imperative to establish cardio-oncology surveillance standards that incorporate NIIS and to encourage clinicians to include NIIS assessment in routine heart-failure follow-up, thereby enabling the early identification of high-risk populations with respect to hospital-detected tumour events at re-admission/follow-up. It is advisable to develop personalized prevention strategies based on the patient’s NIIS level and complementary risk factors—such as age, renal function, New York Heart Association class, C-reactive protein, smoking status, alcohol consumption, *β*-blocker or statin therapy, and nutritional reserves. These strategies should integrate both targeted anti-inflammatory or nutritional interventions and lifestyle modifications to reduce the burden of cancer in heart-failure cohorts.

## Data Availability

The original contributions presented in the study are included in the article/supplementary material, further inquiries can be directed to the corresponding author/s.
